# Synthesis of carbon dots as an antibacterial and antioxidant agent

**DOI:** 10.3389/fchem.2025.1627543

**Published:** 2025-09-08

**Authors:** Ying Zhang, Jie Wang, Xiuhong Wu, Rui Chang, Hongyu Luo, Juan Yang, Quan Wu, Ze Xu, Yingfu Zhong

**Affiliations:** Chongqing Academy of Agricultural Sciences, Chongqing, China

**Keywords:** carbon dots, antibacterial, antioxidant, Yongchuan Xiuya, characterization

## Abstract

**Objective:**

A new type of antibacterial and antioxidant carbon dots has been discovered.

**Methods:**

In this study, a facile one-step hydrothermal method was employed to synthesize carbon dots (CDs) using *Yongchuan Xiuya* as the precursor.

**Results:**

The morphology and chemical composition of the synthesized CDs were systematically characterized. The resulting doped carbon quantum dots (CDs) exhibited a spherical shape with an average particle size of 4.17 nm and a lattice spacing of 0.22 nm. The CDs demonstrated exceptional antioxidant and antibacterial properties, showing significant antimicrobial activity against both Gram-positive (*S. aureus*) and Gram-negative (*E. coli*) bacteria, with minimum inhibitory concentration values of 0.62 mg/mL and 0.85 mg/mL, respectively. Mechanistic studies revealed that bacterial cell death likely results from strong electrostatic interactions between the negatively charged bacterial surfaces and the positively charged CDs.

**Conclusion:**

This work presents a cost-effective and eco-friendly approach to producing carbon dots with dual functionality as both antibacterial and antioxidant agents.

## Graphical Abstract

**Figure:**
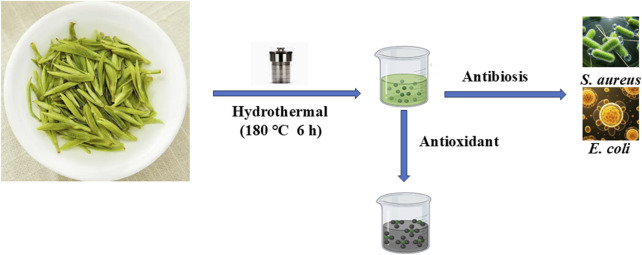

## 1 Introduction

Carbon dots (CDs), a class of zero-dimensional spherical nanoparticles with diameters typically under 10 nm (Li et al., 2023), are characterized by a core-shell structure comprising a sp^2^/sp^3^ hybridized carbon core and a surface decorated with various functional groups (Guo et al., 2022). These nanoparticles can be synthesized from diverse natural carbon precursors (Christopoulou et al., 2021) through a range of methodologies. CDs are renowned for their exceptional optical properties, including a wide emission spectrum within the visible range, high aqueous solubility, remarkable biocompatibility, and robust photostability (Chu et al., 2022; Jiang et al., 2022; Lin et al., 2022; Qu et al., 2022; Wang et al., 2022). Over the past decades, CDs have gained prominence as versatile nanomaterials for applications such as antibacterial agents and fluorescent sensors, attributed to their superior water solubility, stable fluorescence, minimal toxicity, and excellent biocompatibility (Ge et al., 2022; Wang et al., 2020; S¸ enol and Onganer, 2022; Fu et al., 2022; Chai et al., 2021). Recent studies have highlighted the potential of CDs as effective antibacterial agents, leveraging a synergistic interplay of multiple mechanisms to exert their antimicrobial effects (Nie et al., 2020; Pei et al., 2025; Zhao et al., 2022). Among these, the generation of reactive oxygen species (ROS)—including singlet oxygen (^1^O_2_), superoxide (O_2_^−^), hydroxyl radicals (⋅OH), and hydrogen peroxide (H_2_O_2_) (Nie et al., 2020; Zhao et al., 2022; Chen et al., 2022)—plays a pivotal role in inducing oxidative stress, a primary antibacterial mechanism of CDs (Lian et al., 2024; Wang et al., 2021). Recent studies have developed fluorescent probes with low inhibitory concentrations against *E. coli* and *S. aureus*. Among these, honey-derived carbon dots demonstrated the strongest antibacterial activity, exhibiting the lowest MIC and MBC (1.8 mg/mL) against both strains (Mohammadi et al., 2024). The methanol extract of *S. maritima* exhibited antimicrobial activity against *E. coli* (MIC = 25 mg/mL) and *S. aureus* (MIC = 6.25 mg/mL) (Can Aytar, 2024). Date syrup polyphenols exhibited antibacterial activity, with MIC values of 30 mg/mL for *E. coli* and 20 mg/mL for *S. aureus* (Taleb et al., 2016).

The escalating prevalence of bacterial infections, particularly those caused by antibiotic-resistant strains, has become a critical global health challenge, exacerbated by the misuse of antibiotics and the rapid evolution of bacterial resistance (Liu et al., 2022). This underscores the urgent need for innovative strategies to address bacterial infections. Green tea (Camellia sinensis), a member of the Theaceae family, is one of the most widely consumed beverages globally, valued for its numerous health benefits (Atak et al., 2024). *Yongchuan Xiuya*, a premium needle-shaped green tea cultivated in Yongchuan District, Chongqing, is particularly notable for its rich content of essential minerals such as potassium, calcium, magnesium, and manganese, among others (Shuai et al., 2022). This tea is celebrated for its ability to promote sodium excretion, prevent dental caries, and exert antioxidant and anti-aging effects (Tang et al., 2023).

In this study, we synthesized high-quantum-yield CDs using *Yongchuan Xiuya* as the carbon source via a hydrothermal method. The chemical composition and structural properties of the resulting CDs were thoroughly characterized. The synthesized CDs demonstrated significant antibacterial activity against both *E. coli* and *S. aureus*, highlighting their potential as novel antibacterial nanomaterials. This work provides a foundational framework for further exploration of CDs in combating bacterial infections and advancing nanomaterial-based therapeutic strategies.

## 2 Experimental section

### 2.1 Materials

All chemicals and reagents employed in this study were of analytical grade and compliant for experimental use. *Yongchuan Xiuya* tea was procured from a local supermarket in Chongqing, China. Deionized water was used throughout the experiments to ensure consistency and purity. Horseradish peroxidase (HRP) was obtained from Sinopharm Chemical Reagent Co., Ltd. Phosphate buffer solution (PBS) was sourced from Merck (Darmstadt, Germany). Hydroxypropyl acrylate (HPA) and 3-(4,5-Dimethyl-2-Thiazolyl)-2,5-Diphenyl Tetrazolium Bromide (MTT) were supplied by Aladdin Co., Ltd. All materials were used as received without further purification.

### 2.2 Preparation of CDs

The CDs were synthesized via a one-step hydrothermal method. Briefly, 0.20 g of *Yongchuan Xiuya* tea was dispersed in 15 mL of deionized water under stirring to ensure homogeneity. The mixture was then transferred into a 25 mL Teflon-lined autoclave and subjected to hydrothermal treatment at 200 °C for 10 h. After the reaction, the autoclave was allowed to cool to room temperature naturally. The resulting suspension was centrifuged at 8,000 rpm for 10 min to remove large particles. The supernatant was further filtered through a 0.22 μm microporous membrane to obtain a clear solution. To purify the CDs, the solution was dialyzed using a dialysis bag with a molecular weight cutoff of 500 Da for 12 h to remove any unreacted precursors or small impurities. Finally, the purified CDs solution was freeze-dried to yield a solid powder, which was stored at 4 °C for subsequent characterization and applications.

### 2.3 Characterization

Transmission electron microscopy (TEM) images were acquired using a JEOL 2100F instrument (Tokyo, Japan) to analyze the morphology and size distribution of the synthesized CDs. Fourier-transform infrared (FT-IR) spectra were recorded on a Nicolet iS5 spectrometer (Thermo Fisher Scientific Inc., Waltham, MA, USA) to identify the functional groups present on the surface of the CDs. For detailed elemental composition analysis, X-ray photoelectron spectroscopy (XPS) was performed using an ESCALAB Xi + spectrometer (Thermo Fisher Scientific Inc., Waltham, MA, USA). The minimum inhibitory concentration (MIC) assay was conducted using a microplate reader from Awareness Technology (Florida, USA), with absorbance measurements taken at 600 nm. Additionally, the zeta potentials of both the CDs and bacterial cells were determined using a Nano Brook 90Plus Zeta Potential Analyzer (Malvern Instruments, UK) to assess surface charge characteristics. A relative quantum yield (QY) of CDs was determined with quinine sulfate (dispersed in 0.1 M H_2_SO_4_, QY = 54%) as a reference.

### 2.4 Culture of bacterial

*E. coli* and *S. aureus* were cultured in Luria-Bertani (LB) broth medium under standard conditions. Each bacterial strain was inoculated into 5 mL of LB medium and incubated at 37 °C for 12 h with continuous shaking at 180 rpm to ensure optimal growth. The bacterial concentration was monitored by measuring the optical density at 600 nm (OD_600_), and cultures were maintained within an OD600 range of 0.6–0.8 to ensure consistent growth phases. The bacterial suspensions were then diluted to a final concentration of 1.5 × 10^7^ colony-forming units per milliliter (CFU/mL). Following activation, the bacterial suspensions were stored at 4 °C in a refrigerator for subsequent experimental use.

### 2.5 Antibacterial test

The minimum inhibitory concentration (MIC) of CDs against *E. coli* and *S. aureus* was determined using the broth microdilution method in 96-well plates. Briefly, 100 μL of bacterial suspension (1.5 × 10^7^ CFU/mL) was mixed with 100 μL of varying concentrations of CDs in the wells. The plates were then incubated at 37 °C for 12 h. After incubation, bacterial growth was quantified by measuring the optical density at 600 nm (OD_600_). To further assess cell viability, 10 μL of MTT (3-(4,5-dimethylthiazol-2-yl)-2,5-diphenyltetrazolium bromide) solution was added to each well, and the plates were incubated for an additional 20 min. All experiments were performed in triplicate to ensure reproducibility.

To evaluate the potential development of bacterial resistance, the initial MIC values of CDs against *E. coli* and *S. aureus* were determined. Bacteria from the 1/2 MIC concentration were then diluted to 1 × 10^7^ CFU/mL and subjected to a 24-h incubation at 37 °C. This process was repeated daily for 14 consecutive passages, with the MIC values of the CDs-bacteria mixtures recorded at each passage. This longitudinal study aimed to monitor any changes in bacterial susceptibility to CDs over time, providing insights into the potential for resistance development.

### 2.6 Antibacterial mechanism of CDs

Zeta potential measurements were performed to evaluate the surface charge characteristics of the CDs and their interactions with bacterial cells. A diluted CDs solution and a mixture of the diluted CDs solution with activated bacterial suspension were analyzed using a zeta potential analyzer. This analysis provided insights into the electrostatic interactions between the CDs and bacterial surfaces, which are critical for understanding their antibacterial mechanisms.

### 2.7 Antioxidant test

Antioxidant activity was evaluated by DPPH free radical scavenging capacity, and ABTS free radical scavenging capacity comparing with the equivalent antioxidant capacity (TEAC) of 6-hydroxy-2,5,7,8-tetramethylchroman-2-carboxylic acid (Trolox) and the ferric reducing antioxidant power (FRAP).

#### 2.7.1 DPPH free radical scavenging activity

**FIGURE 1:**
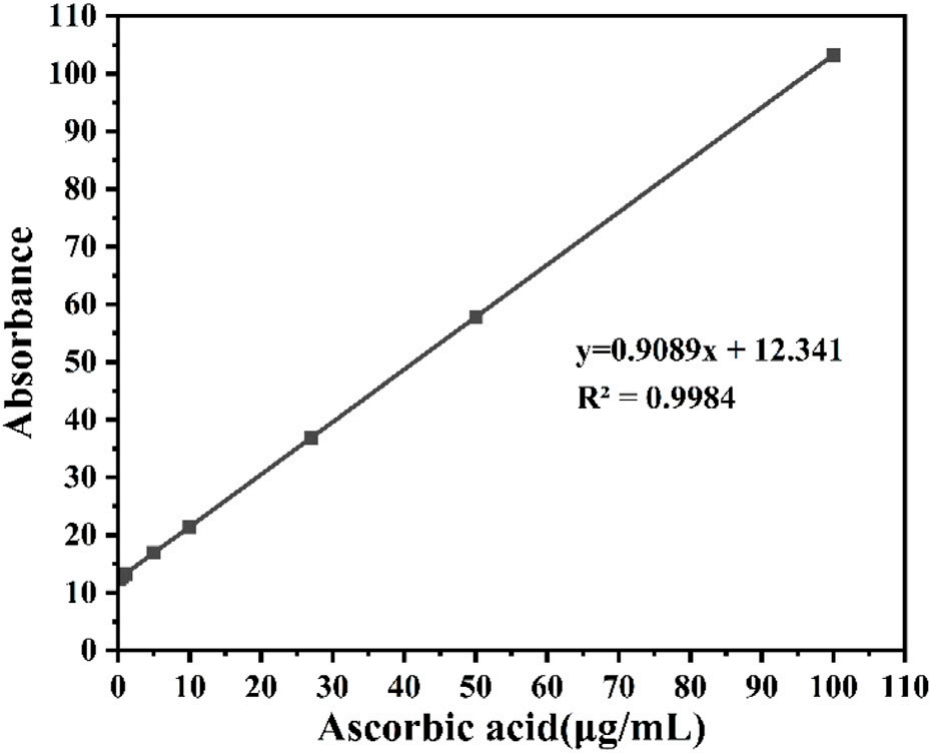
Standard curve of antioxidant test.

The DPPH free radical scavenging activity was measured according to Ghosh et al.'s protocol. Briefly, 200 µL of CDs was mixed with 2,800 µL of 0.2 mM DPPH methanolic solution. After vigorous shaking, the mixture was incubated in the dark at room temperature for 25 min. Absorbance was recorded at 517 nm using a UV–Vis spectrophotometer, with methanol as the control. Ascorbic acid was used as a standard scavenger at concentrations of 0.1, 0.5, 1, 5, 10, 27, 50, and 100 μg/mL. A calibration curve was constructed with the equation *R* ^2^ = 0.9984; y = 0.9089x+12.341, where *y* represents the absorbance and *x* represents the concentration of ascorbic acid, the standard curve is shown in Figure 1. The DPPH scavenging percentage was calculated using the formula:

$$\text{Free radical scavenging }\%=\left[\left(\text{Control}-\text{Sample}\right)/\text{Control}\right]\times 100$$

This method provided a quantitative measure of the CDs’ ability to scavenge free radicals, reflecting their antioxidant potential.

#### 2.7.2 Ferric reducing antioxidant power (FRAP)

The antioxidant capacity assay for CDs was evaluated by the ferric reducing antioxidant power (FRAP). The FRAP reagent is a solution composed of 25 mL of 0.3 mM acetate buffer (pH 3.6), 2.5 mL of 10 mM 2,4,6-Tris (2-pyridyl)-- s-triazine (TPTZ) dissolved in 40 mM hydrochloric acid, and 2.5 mL of 20 mM iron (III) chloride. Aliquots of FRAP solution (270 μL) and each compound diluted in 100% methanol (30 μL) were added to a microplate. Absorbance was read at 595 nm in a microplate reader after incubation (30 min/20 °C). Trolox (0–300 mmol) was used to create the calibration curve. The ferric reducing antioxidant potential was expressed as equivalent mmol of Trolox for each mmol of the compound tested.

### 2.8 Cell viability assay

Evaluation of Synthetic CDs’ Protective Effects Against H_2_O_2_-Induced Cell Damage. The cytoprotective effects of synthetic CDs against H_2_O_2_-induced oxidative stress were analyzed using the CCK-8 assay. First, the cytotoxicity of synthetic peptides on HepG2 cells was tested. Cells were seeded in a 96-well plate (1 × 10^4^ cells/well) and cultured for 24 h at 37 °C. After replacing the medium with fresh medium containing varying peptide concentrations, the cells were incubated for an additional 12 h. The medium was then replaced with 100 μL of fresh medium containing 10 μL of CCK-8 reagent, followed by a 2 h incubation. Absorbance was measured at 450 nm. For oxidative stress protection assessment, HepG2 cells were pretreated with peptides for 12 h before exposure to 175 μmol/L H_2_O_2_ for 2 h. All experiments were conducted in triplicate, and cell viability was calculated as a percentage relative to the control group.

### 2.9 Measurement of ROS activity

HepG2 cells from the control, model and experimental groups were collected, lysed and centrifuged at 12,000 × *g* for 10 min at 4 °C. The supernatants were collected and analyzed using commercial assay kits following the manufacturer’s instructions. ROS levels were quantified using a fluorescence-based assay.

## 3 Results and discussion

### 3.1 Characterization of CDs

**FIGURE 2:**
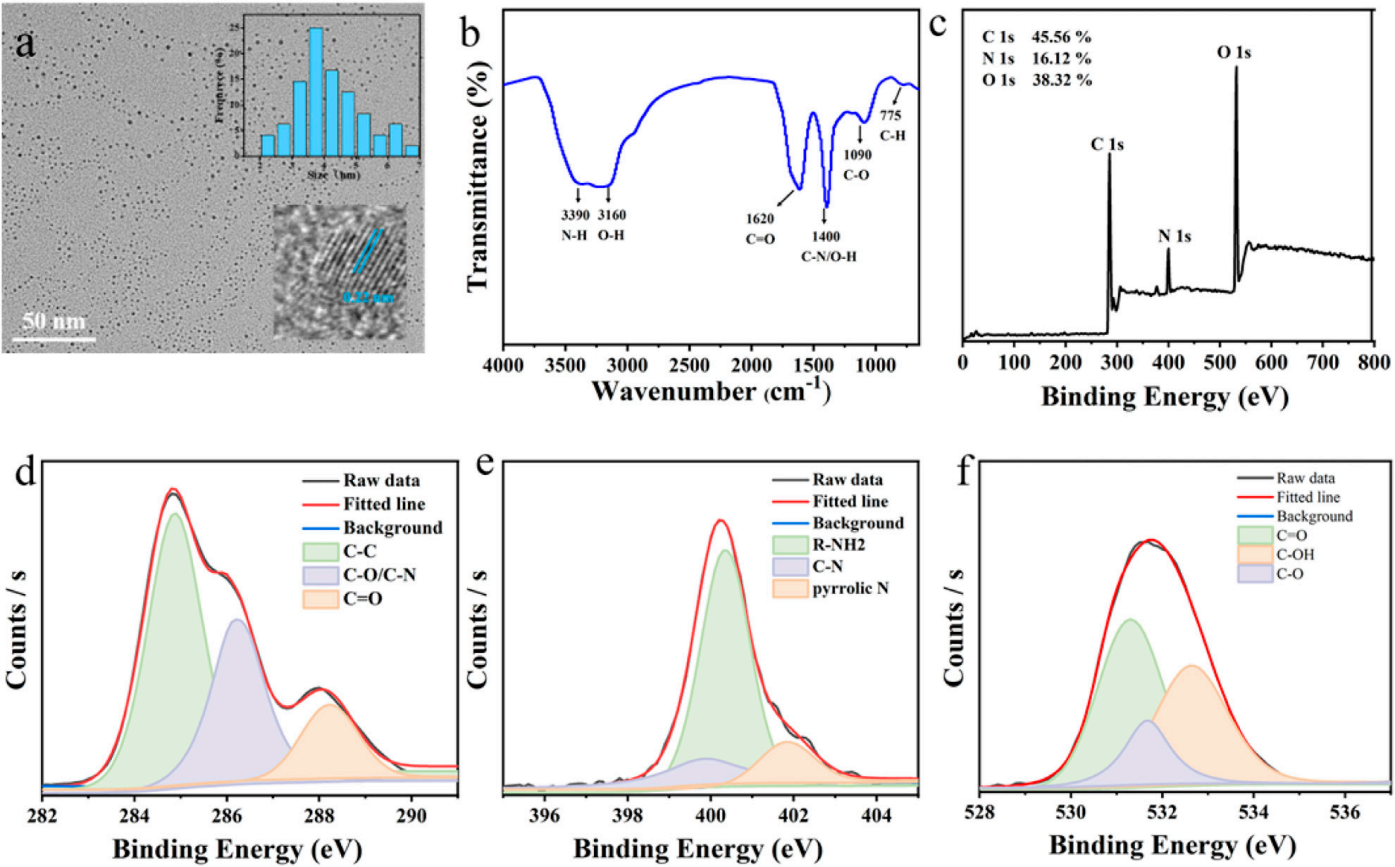
**(a)**TEM image of the CDs and the insets show the particle size distribution and the HRTEM images. **(b)** FTIR spectrum of CDs. **(c)** XPS survey spectrum of CDs. High-resolution XPS spectra of **(d)** C1s, **(e)** N1s, **(f)** O1s.

The morphology and structural characteristics of the synthesized CDs were analyzed using transmission electron microscopy (TEM). As shown in Figure 2a, the CDs exhibited a particle size distribution ranging from 0.21 to 7.38 nm, with an average diameter of approximately 4.17 nm. High-resolution TEM (HRTEM) revealed a well-defined lattice spacing of 0.22 nm, indicating the crystalline nature of the CDs.

To further investigate the surface composition and functional groups of the CDs, Fourier-transform infrared (FT-IR) spectroscopy and X-ray photoelectron spectroscopy (XPS) were employed. The FT-IR spectrum (Figure 2b) displayed characteristic peaks at 3,390 cm^-1^ and 3,160 cm^-1^, corresponding to the stretching vibrations of N–H and O–H groups, respectively. A peak at 1,620 cm^-1^ confirmed the presence of C=O/C=C bonds, while the peak at 1,400 cm^-1^ was attributed to C–N/O–H vibrations. Additionally, a peak at 775 cm^-1^ was associated with C–H stretching.

XPS analysis (Figure 2c) revealed the elemental composition of the CDs, with C 1s (45.56%), O 1s (38.38%), and N 1s (16.12%) being the primary components. The high-resolution C 1s spectrum (Figure 2d) showed peaks at 283.8 eV and 286 eV, corresponding to C=O, C–N, and C=C bonds. The N 1s spectrum (Figure 2e) exhibited peaks at 397 eV, 399.8 eV, and 400 eV, assigned to R–NH_2_, C–N–C, and N–H groups, respectively. The O 1s spectrum (Figure 2f) displayed peaks at 531.2 eV, 532.5 eV, and 534.7 eV, which were attributed to C=O, C–OH, and C–O bonds, respectively. Through elemental analysis, the elemental composition of this carbon dot was determined as follows: C: 44.73%, H: 6.12%, N: 15.35%.

Taking quinoline sulfate as a reference (Jiang et al., 2015), the quantum yield was 18.3%. These results collectively provide a comprehensive understanding of the structural and compositional properties of the synthesized CDs, highlighting their potential for various applications.

### 3.2 Antibacterial activity of CDs

The minimum inhibitory concentration (MIC) of the CDs was determined using a 96-well cell culture plate and the microdilution method. Bacterial suspensions of *E. coli* and *S. aureus* were treated with varying concentrations of CDs and incubated for 12 h under consistent conditions. Bacterial viability was assessed by measuring the optical density at 600 nm (OD_600_) and comparing the values across different treatment groups.

**FIGURE 3:**
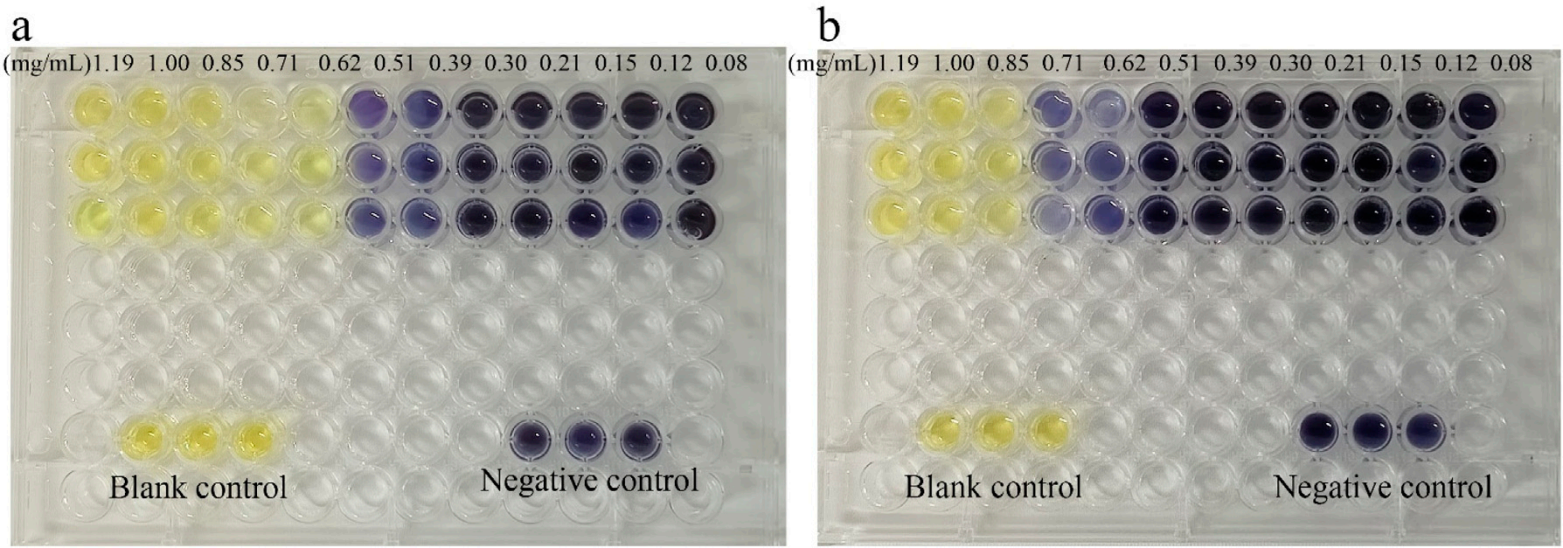
The antibacterial ability of CDs on **(a)** *Escherichia coli* and **(b)** *Staphylococcus aureus*.

As illustrated in Figures 3a,b, the viability of both *E. coli* and *S. aureus* decreased progressively with increasing concentrations of CDs, demonstrating a dose-dependent antibacterial effect. The MIC values, defined as the lowest concentration of CDs that completely inhibited bacterial growth, were determined to be 0.62 mg/mL for *E. coli* and 0.85 mg/mL for *S. aureus*. These results highlight the potent antibacterial activity of the CDs against both Gram-negative and Gram-positive bacteria, underscoring their potential as effective antimicrobial agents. However, the extract of *Yongchuan Xiuya* did not show excellent antibacterial effects. This indicates that after converting *Yongchuan Xiuya* into carbon dots, the antibacterial activity was enhanced.

As summarized in Table 1, numerous studies have successfully synthesized fluorescent probes capable of inhibiting both Gram-positive and Gram-negative pathogens at low concentrations. Notably, carbon dots exhibit significantly enhanced antibacterial activity compared to other tested compounds.

**TABLE 1:** Comparison of different quantification methods to *Staphylococcus aureus* and *Escherichia coli.*

Methods	MIC_*(S. aureus)*_	MIC_*(E. coli)*_	References
N-CQDs/TNP	20 mg/L	20 mg/L	Zheng et al. (2024)
DS	20 mg/mL	30 mg/mL	Das et al. (2024)
Liangguoan	20 mg/mL	40 mg/mL	Zeng et al. (2021)
GEO	1 mg/mL	2 mg/mL	Wang et al. (2023)

**FIGURE 4:**
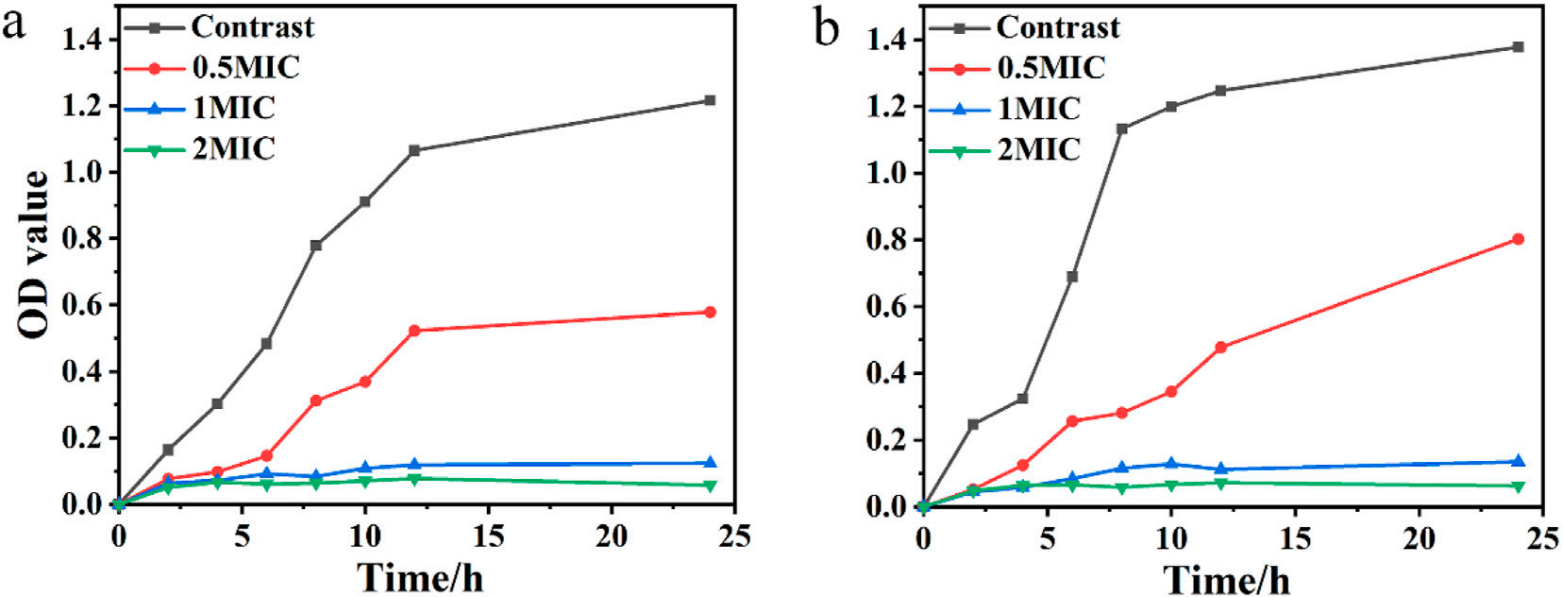
Inhibitory effect on **(a)** *Escherichia coli* and **(b)** *Staphylococcus aureus* of different concentrations of CDs.

The time-dependent antibacterial activity of the CDs was evaluated by monitoring the optical density at 600 nm (OD600) over time, as shown in Figures 4a,b. The results demonstrated that the antibacterial efficacy of the CDs was strongly concentration-dependent, with higher concentrations of CDs leading to a more significant reduction in bacterial growth. Notably, when treated with CDs at a concentration equivalent to 1× the minimum inhibitory concentration (MIC), the growth of both *E. coli* and *S. aureus* was completely inhibited within 24 h. This finding underscores the potent and rapid antibacterial action of the CDs, highlighting their potential as effective agents for controlling bacterial infections.

### 3.3 Antibacterial mechanism of CDs

**FIGURE 5:**
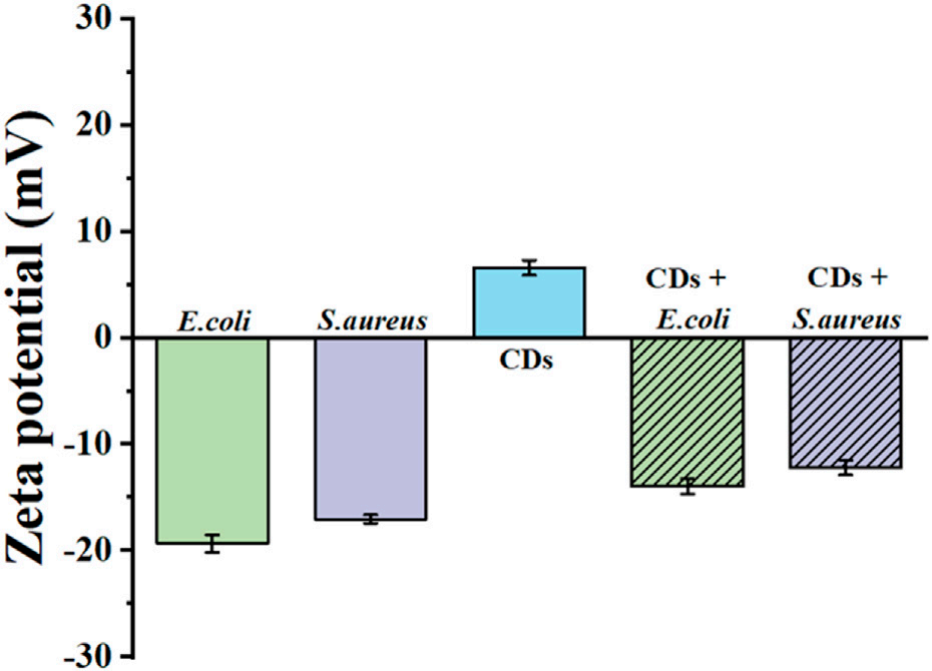
Zeta potential distribution of CDs.

To further investigate the antibacterial mechanism of the CDs, the zeta potential of the CDs was examined. As shown in Figure 5, the zeta potentials of both the CDs and the bacterial cells were measured. The zeta potentials of *E. coli* and *S. aureus* were determined to be −19.63 mV and −26.76 mV, respectively, reflecting the negatively charged nature of their cell walls. In contrast, the CDs exhibited a positive zeta potential because of the presence of cationic groups such as amino groups, facilitating their electrostatic adsorption onto the negatively charged bacterial surfaces. This interaction is a critical step in the antibacterial activity of the CDs, as it enables close contact between the CDs and the bacterial cells.

### 3.4 Antioxidant activity of CDs

The antioxidant activity of the CDs was evaluated using three different *in vitro* methods, DPPH (2,2-diphenyl-1picrylhydrazyl) radical scavenging and ferric reducing antioxidant power (FRAP) assay.

The objective of this experiment was to evaluate the free radical scavenging activity of the CDs using the 2,2-diphenyl-1-picrylhydrazyl (DPPH) assay. The results demonstrated that the CDs possess significant antioxidant potential, with a DPPH radical scavenging activity of 91.32% ± 0.31%. This high scavenging efficiency highlights the strong ability of the CDs to neutralize free radicals, underscoring their potential as effective antioxidant agents. These findings suggest that the CDs could be valuable in applications requiring oxidative stress mitigation, such as in biomedical or environmental contexts.

In the FRAP assay, an antioxidant reduces Fe^3+^ to Fe^2+^ by donating electrons. The reducing power of the synthesized compounds measured using the FRAP test revealed that CDs has the best-reducing capacity. The results demonstrated that the CDs possess significant antioxidant potential, with FRAP radical scavenging activity of 1,320% ± 32%.

### 3.5 CDs protect HepG2 cells from H_2_O_2_-induced oxidative damage

**FIGURE 6:**
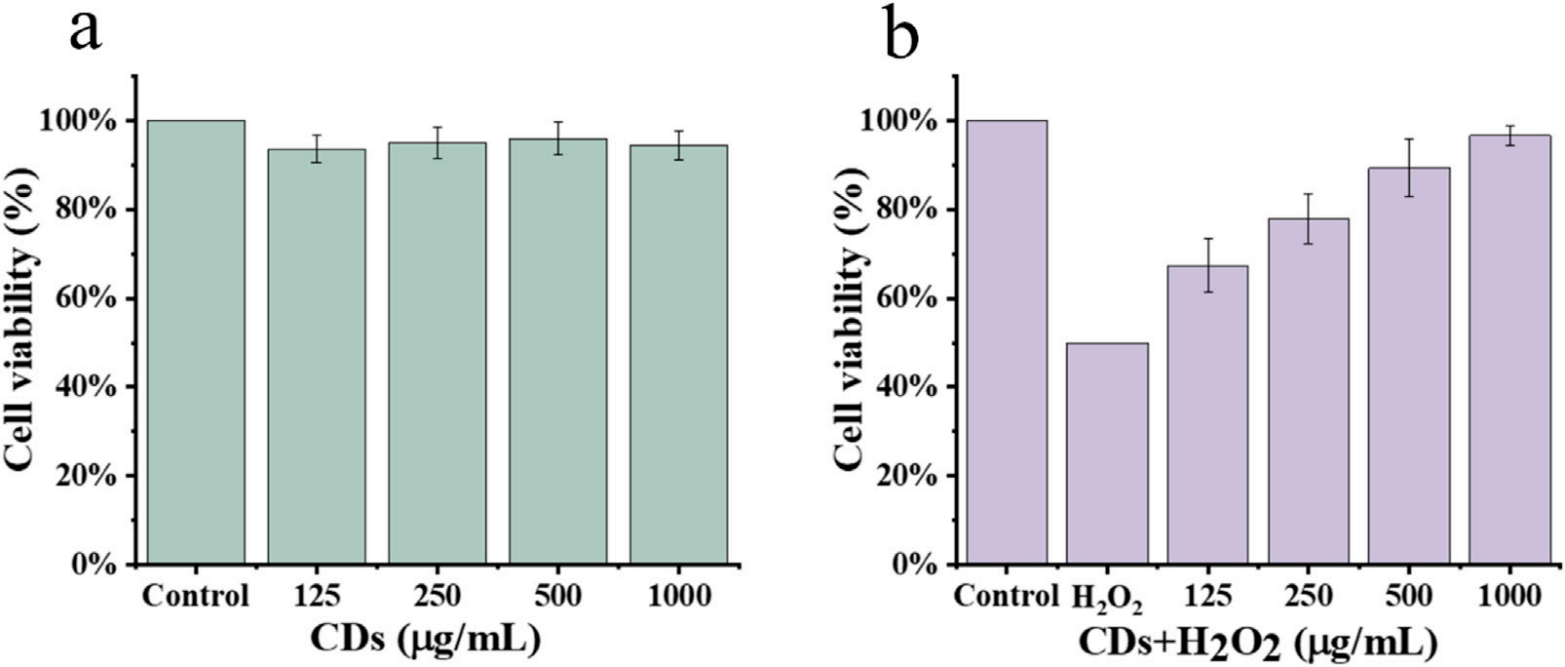
Protective effects against oxidative stress of CDs. Cytotoxicity of the CDs **(a)** and its cytoprotective effect in H_2_O_2_- induced oxidative stress **(b)**.

HepG2 cells, derived from human hepatocellular carcinoma, are widely used for antioxidant assay of peptides *in vitro*. Therefore, this study employed the HepG2 cell model to evaluate the antioxidant activity of CDs. Initially, we assessed the cytotoxicity of the CDs at concentrations of 125, 250, 500, and 1,000 μg/mL. As shown in Figure 6a, the cell viability remained above 90% at all tested concentrations, indicating that the CDs exhibited no significant cytotoxicity towards HepG2 cells at these concentrations. Therefore, the concentrations used in this study are considered safe and reasonable. Subsequently, HepG2 cells were pre-incubated with the CDs and H_2_O_2_ was used to simulate oxidative stress (Figure 6b). The results showed that exposure to H_2_O_2_ significantly decreased cell viability (*p <* 0.05) compared to the control group, indicating that oxidative stress induced cellular damage. In contrast, pre-incubation with CDs significantly improved cell viability in a concentration-dependent manner, demonstrating their protective effect against H_2_O_2_-induced oxidative damage.

The antioxidant effect of carbon dots can last for more than 3 days and can be explained by the hydrogen donor groups present on their surface (such as hydroxyl (-OH) and carboxyl (-COOH)). The conjugated sp^2^ carbon structure inside the carbon dots enables the delocalization of electrons, which is conducive to the formation of free radical intermediates. The interaction between the free radicals and the functional groups on the carbon dots leads to the formation of stable free radical adducts, effectively neutralizing reactive oxygen species (ROS). Due to the stability of the sp^2^ carbon core and the diversity of the surface functional groups, carbon dots can maintain their antioxidant activity for a long time.

## 4 Conclusion

In summary, CDs were successfully synthesized via a hydrothermal method by *Yongchuan Xiuya*. Using *S. aureus* and *E. coli* as model bacteria, the CDs exhibited remarkable antioxidant and antibacterial activities. The antibacterial mechanism study revealed that bacterial cell death is likely attributed to the strong electrostatic interactions between the negatively charged bacterial surfaces and the positively charged CDs. This study presents the development of a cost-effective and environmentally friendly carbon dot material, which has been proven to possess dual functionality as both an antioxidant and an antibacterial agent.

## Data Availability

The original contributions presented in the study are included in the article/supplementary material, further inquiries can be directed to the corresponding authors.
